# Effects of Altered Maternal Folic Acid, Vitamin B_12_ and Docosahexaenoic Acid on Placental Global DNA Methylation Patterns in Wistar Rats

**DOI:** 10.1371/journal.pone.0017706

**Published:** 2011-03-10

**Authors:** Asmita Kulkarni, Kamini Dangat, Anvita Kale, Pratiksha Sable, Preeti Chavan-Gautam, Sadhana Joshi

**Affiliations:** Department of Nutritional Medicine, Interactive Research School for Health Affairs, Bharati Vidyapeeth University, Pune, India; Rikagaku Kenkyūsho Brain Science Institute, Japan

## Abstract

Potential adverse effects of excess maternal folic acid supplementation on a vegetarian population deficient in vitamin B_12_ are poorly understood. We have previously shown in a rat model that maternal folic acid supplementation at marginal protein levels reduces brain omega-3 fatty acid levels in the adult offspring. We have also reported that reduced docosahexaenoic acid (DHA) levels may result in diversion of methyl groups towards DNA in the one carbon metabolic pathway ultimately resulting in DNA methylation. This study was designed to examine the effect of normal and excess folic acid in the absence and presence of vitamin B_12_ deficiency on global methylation patterns in the placenta. Further, the effect of maternal omega 3 fatty acid supplementation on the above vitamin B_12_ deficient diets was also examined. Our results suggest maternal folic acid supplementation in the absence of vitamin B_12_ lowers plasma and placental DHA levels (p<0.05) and reduces global DNA methylation levels (p<0.05). When this group was supplemented with omega 3 fatty acids there was an increase in placental DHA levels and subsequently DNA methylation levels revert back to the levels of the control group. Our results suggest for the first time that DHA plays an important role in one carbon metabolism thereby influencing global DNA methylation in the placenta.

## Introduction

In developing countries, micronutrient deficiencies such as folic acid and vitamin B_12_ are common and associated with poor pregnancy outcomes, having long term health effects. The peri or post-conceptional period represents a sensitive window during which suboptimal maternal micronutrients may affect feto-placental development [1]. In view of this, maternal folic acid supplementation is in operation for the last few decades in India. This is regardless of the fact that there is still a poor knowledge about potential adverse effects on a population mainly consuming a vegetarian diet that may lack vitamin B_12_ [2]. Although early results from the fortification policy indicate beneficial effects in terms of reduction in plasma homocysteine [3]–[5], recent reports suggest adverse effects in humans [6], [7]. During growth high folic acid administration has been shown to alter dietary protein metabolism and to decrease fetal size, as compared to rats fed a control diet [8]. It has also been reported recently, that high folate intakes in vitamin B_12_ deficient mothers are shown to increase the risk of type 2 diabetes in the offspring suggesting that defects in one-carbon metabolism might be at the heart of intrauterine programming of adult disease [9].

Our earlier studies in the rat model that maternal folic acid supplementation at marginal protein levels reduces the levels of brain essential polyunsaturated fatty acid levels especially omega 3 fatty acids in the offspring [10], [11]. Ongoing studies in our lab have also highlighted the importance of docosahexaenoic acid (an omega 3 fatty acid) during pregnancy [12], [13]. Docosahexaenoic acid (DHA) is an indispensable component of all cell membranes and is incorporated in high concentrations in the membrane phospholipids of brain and retina [14] and its availability during the perinatal period is shown to be associated with long term cognitive and visual development [15], [16].

**Figure 1:**
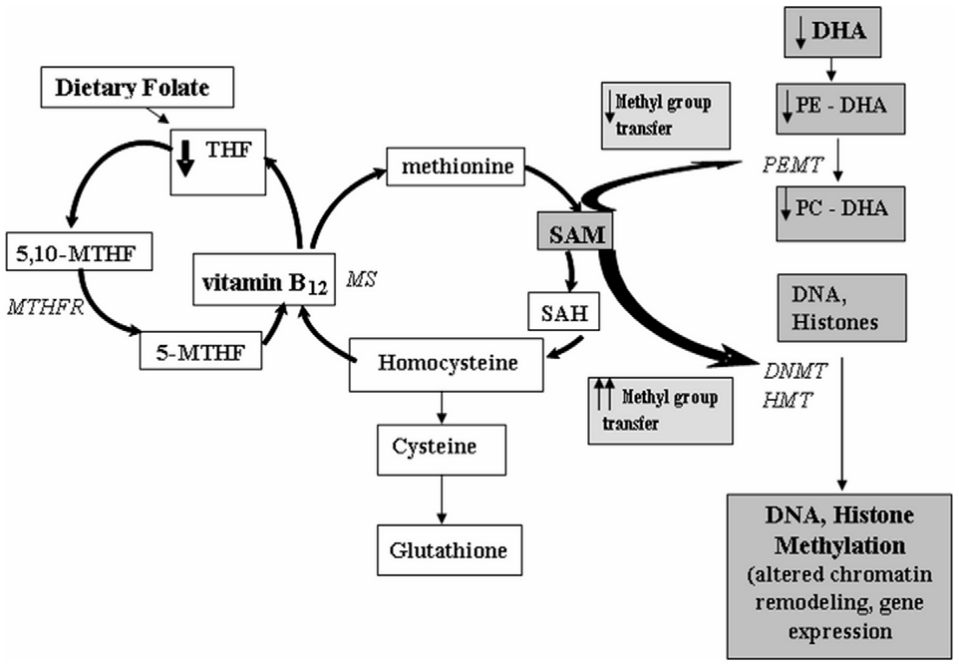
One-Carbon Cycle: Interactions of folic acid, vitamin B_12_ and DHA. THF- tetrahydrofolate; 5, 10-MTHF- 5, 10-methylenetetrahydrofolate; 5-MTHF- 5-methyltetrahydrofolate; MTHFR- methylenetetrahydrofolate reductase; MS- methionone synthase; SAH- S-adenosylhomocysteine; SAM- S-adenosylmethionine; DHA-docosahexanoic acid; PE-DHA- phosphatidylethanolamine-DHA; PC-DHA-phosphatidylcholine-DHA; PEMT- phosphatidylethanolamine-N-methyltransferase; DNMT- DNA methyltransferase; HMT- Histone methyltransferase; ↓- reduced; ↑↑-increased.

It is well established that folate and vitamin B_12_ are the major determinants of one carbon metabolism in which S-adenosyl methionine (SAM) a methyl group donor is formed [17]. Dietary folate is converted in the body to 5 methyl tetrahydrofolate (5-MTHF) by the enzyme methylene tetrahydrofolate reductase (MTHFR). The transfer of methyl group from 5-MTHF to homocysteine requires vitamin B_12_ and results in the synthesis of methionine. Methionine is the precursor for SAM. Methyl groups from SAM are transferred by phosphatidyl ethanolamine–N–methyltransferase (PEMT) to DHA and to DNA and histones by the respective methyltransferases. Phosphatidylcholine (PC) is critical for the delivery of important polyunsaturated fatty acids (PUFA) such as docosahexaenoic acid from the liver to the plasma and distribution to peripheral tissues. Fig. 1 shows the interactions of folic acid, vitamin B_12_ and DHA. We have recently described that when DHA levels are low, there will be less methyl group requirement for conversion of PE-DHA to PC-DHA and may result in excess methyl group availability for other trans-methylation reactions such as DNA and histone methylation leading to altered chromatin remodeling and gene expression [18]. We therefore hypothesize that omega 3 fatty acids play a key role in one carbon metabolism affecting global methylation levels.

Placenta is an organ whose proper development and function are crucial to the health, growth, and survival of the developing fetus. A number of studies are now making important links between alterations to appropriate epigenetic regulation in the placenta and diseases of gestation and early life [19]. Examining epigenetic alterations in the placenta will prove especially important in the search for biomarkers of exposure, pathology, and disease risk and can provide critical insights into the biology of development and pathogenesis of disease [20].

The present study therefore for the first time, examines the effect of normal and excess folic acid in the absence and presence of vitamin B_12_ deficiency on global methylation patterns in the placenta. Further, the effect of maternal omega 3 fatty acid supplementation on the above vitamin B_12_ deficient diets was also examined.

## Materials and Methods

This study was carried out in accordance with the CPCSEA guidelines (Committee for the purpose of control and supervision of experimental animals) Govt of India. This study was approved by the Bharati Vidyapeeth Animal Ethical Committee (IAEC/CPCSEA/258). The institute is recognized to undertake experiments on animals as per the CPCSEA, Govt of India.

### Animals

Wistar albino rats (60F, 20M) of average weight 150 g were obtained from National Toxicology Center animal house. Instead of using them directly for the experimental protocol it was thought appropriate to use their progeny. They were maintained at 22°C on a controlled 12-hr light and 12 hr dark cycle with appropriate ventilation system. Animals were marked with picric acid as H (head), Back (B), Tail (T) etc for identification.

### Breeding

These pups were then put for breeding at 3 months of age. Males were housed individually prior to mating to acquire cage dominance. Virgin female rats were allowed to breed (sex ratio 1:3). On the following morning the vaginal smears were taken to confirm mating. Vaginal smears were taken on a clean microscope slide using a cotton bud dipped in saline. The slides were examined under a microscope at 10× magnification. The sperm positive smear was considered a result of successful mating and considered day 0 of gestation. The pregnant dams were housed individually (in polypropylene cages of 29×22×14 cm dimensions containing rice husk as bedding material). Animals receiving Vitamin B_12_ deficient diets were kept in special cages to prevent coprophagy.

Out of 60 females, 47 females became pregnant and were divided randomly into 6 dietary groups. All dams were delivered by C section on day 20 of gestation.

### Diets

The composition of the control and the treatment diets (Table 1) was as per AIN 93 purified diets for laboratory rodents [21]. Protein level in the control and treatment diets was 18%. Total of six isocaloric treatment diets were formulated and has been described by us recently [22], [23]. Briefly four diets were formulated for examining the effects of 2 different levels of folic acid (i.e 2 and 8 mg folic acid/kg diet) during pregnancy both in the presence and absence of vitamin B_12_. In addition, 2 more diets were formulated to examine the effects of omega 3 fatty acid (DHA+EPA (Eicosapentaenoic acid)) supplementation on both the vitamin B_12_ deficient groups. Vitamin B_12_ deficiency was obtained exclusively through dietary means. Vitamin free casein was used for all treatment diets. Thus there were a total of 6 groups:

Control- normal folate, normal B12, NFBD- normal folate, B12 deficient, NFBDO-normal folate, B12 deficient, omega 3 supplemented, EFB- excess folate, normal B12, EFBD-excess folate, B12 deficient, EFBDO- excess folate, B12 deficient, omega 3 supplemented.

**Table 1:** Composition of the diets.

S.No	Diets	Control (g/kg)	NFBD (g/kg)	EFB (g/kg)	EFBD (g/kg)	NFBDO (g/kg)	EFBDO (g/kg)
1.	Corn Starch	398	398	398	398	398	398
2.	Casein	200	200	200	200	200	200
3.	Dextrinized Starch	132	132	132	132	132	132
4.	Sucrose	100	100	100	100	100	100
5.	Soya Bean Oil	70	70	70	70	25	25
6	Fish oil	0	0	0	0	45	45
7.	Fiber	50	50	50	50	50	50
8.	Mineral mixture*	35	35	35	35	35	35
9	Vitamin mixture†	10	10	10	10	10	10
	Folic acid	0.002	0.002	0.008	0.008	0.002	0.008
	Vitamin B_12_	0.025	0	0.025	0	0	0
10	Cystine	3	3	3	3	3	3
11.	Choline Bitartarate	2.5	2.5	2.5	2.5	2.5	2.5
12.	Tertiary Butyl Hydroquinone	0.014	0.014	0.014	0.014	0.014	0.014
	Total Energy (kJ)	1.57	1.57	1.57	1.57	1.57	1.57

*Mineral mixture (g/kg mixture):Calcium carbonate, 357; Potassium Phosphate, 196; Potassium Citrate, 70.78; Sodium Chloride, 78; Potassium Sulphate, 46.6; Magnesium Oxide, 24; Ferric Citrate, 6.06; Zinc Carbonate, 1.65; Manganous Carbonate, 0.63; Cupric Carbonate, 0.3; Potassium Iodate, 0.01; Sodium Selenate, 0.01; Ammonium Paramolybdate, 0.007; Sodium Metasilicate, 1.45; Chromium Potassium Sulphate, 0.275; Lithium Chloride, 0.01; Boric Acid, 0.08; Sodium Fluoride, 0.06; Nickel Carbonate, 0.03; Ammonium Vanadate, 0.006; Sucrose, 221.02.

† **Vitamin mixture (g/kg mixture):**Nicotinic Acid, 3; Calcium Pantothenate, 1.6; Pyridoxine-HCl, 0.7; Thiamin –HCl, 0.6; Riboflavin, 0.6; D-Biotin, 0.02; Vitamin B_12_ (in 0.1% Mannitol), 2.5;Vitamin E, 15;Vitamin A, 0.8; Vitamin D-3, 0.25;Vitamin K, 0.075;Folic acid, 0.2 (control) and Sucrose 974.655, was used to make total weight of the vitamin mixture to 1 kg.

Control: Normal folate, normal B12, NFBD: normal folate, B12 deficient, NFBDO: normal folate, B12 deficient, omega 3 supplemented, EFB: Excess folate, normal B12, EFBD: Excess folate, B12 deficient, EFBDO: Excess folate, B12 deficient, omega 3 supplemented.

The lowest level, i.e. 2 mg/kg represents the normal level of folic acid used in the control diet as per the current AIN 93 guidelines while 8 mg/kg is roughly 4 times the requirement of a normal rat. This is in accordance with the fact that folic acid requirement for Indian pregnant woman is set at 400 µg/d, which is 4 times the requirement of a non-pregnant woman. The level of omega 3 fatty acid supplementation was chosen to have an omega 6/omega 3 ratio of 1:1 which is considered to be the ideal ratio [24].

### Observations recorded

During pregnancy, dam weights were recorded at day 0, 7, 14 & 20 to obtain weight gains. On day 20 of gestation the litter weight and size was recorded in each group.

### Maternal plasma folic acid, vitamin B_12_ and homocysteine levels

Plasma vitamin B_12_ and plasma folic acid were measured using a radioimmunoassay kit (Diagnostic Products Corporation, USA) [25] and plasma total homocysteine was determined using the IMx System (Abbott Laboratories, IL, USA) [26].

### Tissue collection and processing

Dams were dissected at day 20 of gestation and placental tissues were collected. Fetal membranes were trimmed off and the placenta was weighed. Placentas were snap frozen and stored at −80°C until assayed.

### Placental fatty acid levels

The procedure for fatty acid analysis used in our study was revised from the original method of Manku et al. that has been reported by us earlier in a separate study [27], [28]. Briefly, placental tissue was homogenized with chilled PBS and centrifuged at 10000 rpm at 4°C for 20 min. Supernantant and cell membrane fractions were separated. Transesterification of cell membrane phospholipid fraction was carried out using hydrochloric acid-methanol. These were separated using a Perkin Elmer gas chromatograph (SP 2330, 30 m capillary Supelco column. Helium was used as carrier gas at 1 mL/min. Oven temperature was held at 150°C for 10 min, programmed to rise from 150 to 220°C at 10°C/min, and at 220°C for 10 min. The detector temperature was 275°C and the injector temperature was 240°C. Retention times and peak areas were automatically computed. The column was calibrated by injecting the standard fatty acid mixture in approximately equal proportion. The data was recorded and the peaks were identified as per the retention time of the standard fatty acids (Sigma) run under the identical conditions. Fatty acids were expressed as g/100 g fatty acid. Total of 15 fatty acids were detected. Saturated fatty acids include myristic acid, palmitic acid and stearic acids, while total monounsaturated fatty acids include myristoleic, palmitoleic, oleic acid and nervonic acids. The omega 3 fatty acids included alpha linolenic acid, eicosapentaenoic acid and docosahexaenoic acid while total omega 6 fatty acids included linoleic acid, gamma linolenic acid, di-homo-gammalinolenic acid, docosapentaenoic acid and arachidonic acid.

### Placental global methylation patterns

Genomic DNA extraction from placental tissues was carried out with the Qiagen Blood and Tissue kit. Global DNA methylation was measured using the Methylamp™ Global DNA Methylation Quantification Kit (Epigentek Group Inc., New York, NY, U.S.A.) as we have described recently [29]. The kit yields accurate measures of methylcytosine content as a percentage of total cytosine content.

The methodology for estimation of global methylation levels used in this study takes into account methylation of all CpG's irrespective of their position in the genome (promoter and non-promoter CpG). This is in concurrence with studies indicating that CpG methylation in intragenic and intergenic regions are also critical to gene expression [30].

### Statistical Analysis

Litter means were used as the unit of analysis. Values are mean ± SD. The data were analyzed using SPSS/PC+ package (Version 11.0, Chicago IL). The treatment groups were compared with the control group by ANOVA and the post-hoc least significant difference test.

## Results

### Feed intake

Feed intake during pregnancy was between 15–19 g/day. There was no effect of different levels of folic acid both in the presence and absence of vitamin B_12_ on feed intake. In contrast, feed intake in both the omega 3 fatty acid supplemented groups was lower (p<0.01) than control. Further feed intake in omega 3 fatty acid supplemented groups were also lower than those in their respective B_12_ deficient groups (NFBD Vs NFBDO, p<0.05) and (EFBD Vs EFBDO, p<0.05) and has been reported by us recently [23].

### Reproductive performance

There was no effect of folic acid supplementation in the presence of vitamin B_12_ (EFB) on weight gain of dams as compared to control during pregnancy. There was also no difference in weight gain in the dams fed omega 3 fatty acids as compared to control or any of the other treatment groups. The pup weight between groups was comparable and has been reported by us recently[23].

### Maternal plasma folic acid, vitamin B_12_, homocysteine and fatty acid levels

As expected, folic acid supplementation (EFB and EFBD) increased (p<0.05) plasma folic acid as compared to controls (Table 2). Similarly animals fed a vitamin B_12_ deficient diet had lower (p<0.05) plasma vitamin B_12_ levels as compared to control. Homocysteine concentrations were comparable between groups. (Table 2)

**Table 2:** Dam plasma folate, vitamin B_12_ and homocysteine levels.

	Control (n = 8)		NFBD (n = 8)		EFB (n = 7)		EFBD (n = 8)		NFBDO (n = 7)		EFBDO (n = 6)	
	Mean	SD	Mean	SD	Mean	SD	Mean	SD	Mean	SD	Mean	SD
**Folic acid (ng/ml)**	26.00	14.48	23.50	12.32	69.86 **	8.47	73.88 **	3.36	34.43	16.05	71.00 **	4.43
**Vitamin B_12_ (pg/ml)**	287.63	56.33	192.29**	28.53	274.00	70.58	188.25 ** ‡‡	33.01	182.00 **	17.52	181.00 ** ‡‡ §§	25.63
**Homocysteine (µmoles/L)**	7.67	1.24	7.53	1.42	6.89	1.48	7.24	1.35	8.97	1.34	7.58 ^#^	2.21

**P<0.01 when compared to control;

† *P*<0.05,

†† *P*<0.01 when compared to NFBD;

‡ *P*<0.05,

‡‡ *P*<0.01 when compared to EFB;

§§ *P*<0.01 when compared to EFBD.

AA: Arachidonic acid; Control: Normal folate, normal B_12_, NFBD: normal folate, B_12_ deficient, NFBDO: normal folate, B_12_ deficient, omega 3 supplemented, EFB: Excess folate, normal B_12_, EFBD: Excess folate, B_12_ deficient, EFBDO: Excess folate, B_12_ deficient, omega 3 supplemented.

### Placental fatty acid levels

DHA levels were significantly (p<0.05) reduced in both the NFBD and EFBD groups as compared to control (Table 3). In contrast, supplementation with omega 3 fatty acids improved (p<0.01) DHA and omega 3 fatty acid levels but reduced arachidonic acid and omega 6 fatty acid (p<0.05) levels in NFBDO as well as EFBDO groups. MUFA (mono-unsaturated fatty acid) levels in the NFBDO group and EFBDO were reduced as compared to NFBD and EFBD groups respectively (p<0.01 for both).

**Table 3:** Placental fatty acid levels in different treatment groups.

	Control(n = 15)		NFBD(n = 16)		EFB (n = 16)		EFBD (n = 15)		NFBDO (n = 16)		EFBDO (n = 14)	
	mean	SD	mean	SD	mean	SD	mean	SD	mean	SD	mean	SD
**Alpha linolenic acid**	1.86	4.72	0.62	0.19	0.40 *	0.26	2.13 ‡‡	4.15	1.62	3.75	1.73	4.47
**Linoleic acid**	12.77	1.39	12.01	1.30	12.19	1.73	13.25 †† ‡	1.20	11.51**	1.80	10.89** † ‡‡ §§	0.97
**Docosahexaenoic acid**	3.73	0.88	2.94 *	0.99	3.48	0.64	2.76 ** ‡	0.57	6.75 ** ††	2.17	7.72 ** ‡‡ §§	2.07
**Arachidonic acid**	17.98	2.07	17.29	2.22	17.71	1.76	17.33	2.10	12.85 ** ††	3.35	12.49 ** ‡‡ §§	1.58
**Omega 3 fatty acids**	5.68	4.53	3.68 **	1.09	4.15	0.71	4.98	3.95	9.93 ** ††	3.28	11.68 ** ‡‡ §§	3.76
**Omega 6 fatty acids**	31.86	3.32	30.49	2.90	31.17	2.88	31.60	2.85	27.35 ** ††	4.49	26.97 ** ‡‡ §§	1.82
Monounsaturated fatty acids	13.85	1.80	15.08	1.78	13.25	3.18	15.01 ‡	3.05	12.38 ††	3.49	12.34 §§	2.72
**Saturated fatty acids**	40.61	4.82	43.58	2.39	36.80 *	6.51	41.59 ‡	4.71	44.27 *	4.61	42.97 ‡	4.40

*P<0.05,

**P<0.01 when compared to control;

† *P*<0.05,

†† *P*<0.01 when compared to NFBD;

‡ *P*<0.05,

‡‡ *P*<0.01 when compared to EFB;

§§ *P*<0.01 when compared to EFBD.

Control: Normal folate, normal B_12_, NFBD: normal folate, B_12_ deficient, NFBDO: normal folate, B_12_ deficient, omega 3 supplemented, EFB: Excess folate, normal B_12_, EFBD: Excess folate, B_12_ deficient, EFBDO: Excess folate, B_12_ deficient, omega 3 supplemented.

### Placental global methylation levels

**Figure 2:**
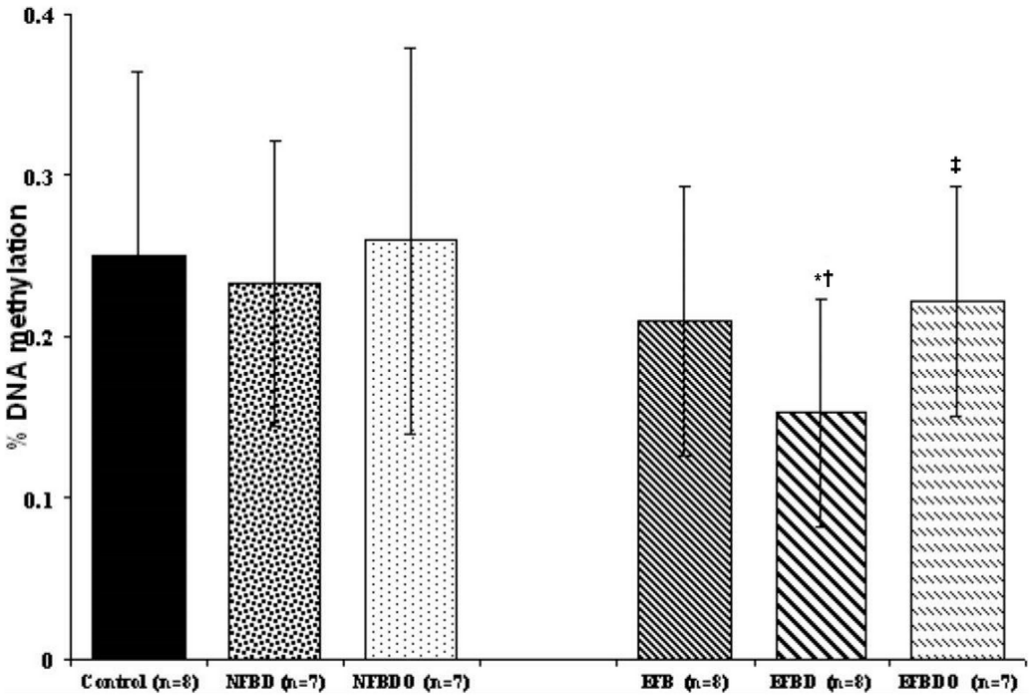
Percent global DNA methylation in Wistar rat placenta. *p<0.05 when compared to control (Normal folate, normal B_12_); ^†^ p<0.05 when compared to NFBD (Normal folate, B_12_ deficient); ^‡^p<0.05 when compared to EFBD (Excess folate, B_12_ deficient), NFBDO: normal folate, B_12_ deficient, omega 3 supplemented, EFB: Excess folate, normal B_12_ ; EFBDO: Excess folate, B_12_ deficient, omega 3 supplemented.

Global DNA methylation levels in placental tissue were reduced in the EFBD group as compared to control and NFBD group (p<0.05). In contrast, in the EFBDO group DNA methylation levels were higher (p<0.05) as compared to the EFBD and were comparable to control (Fig. 2).

## Discussion

This is the first report that has examined the effect of two levels (normal and excess) of folic acid both in presence and absence of vitamin B_12_ deficiency on global DNA methylation levels in the placenta. Our results indicate 1) altered levels of maternal micronutrients did not influence homocysteine concentrations 2) placental DHA levels were reduced in the vitamin B_12_ deficient groups 3) excess maternal folic acid supplementation in the absence of vitamin B_12_ results in reduced global DNA methylation levels 4) when omega 3 fatty acids were supplemented to the diet with excess maternal folic acid and vitamin B_12_ deficiency, DNA methylation levels revert back to the levels observed in the control group.

Our findings for the first time suggest that maternal vitamin B_12_ deficiency, both at normal and excess folic acid levels reduces placental DHA concentrations although mechanisms need to be understood. We and others have previously reported that folic acid alters DHA levels in animals [15], [16]. It may be possible that vitamin B_12_ deficiency either results in a virtual deficiency of folic acid thereby affecting methyl group supply or PEMT may be epigenetically altered leading to reduced expression affecting the conversion of PE-DHA (phosphatidyl ethanolamine-DHA) to PC-DHA (phosphatidyl choline-DHA) resulting in lower DHA levels in placenta. The PC/PE ratio also modulates the activity of Delta-5 and Delta-6 desaturases involved in omega 3 and omega 6 PUFA synthesis [31].

Reports suggest that an imbalance between folate and vitamin B_12_ during pregnancy could influence imprinting in the embryo, perhaps by an effect on DNA methylation since folates are co-factors and co-substrates for biological methylation and nucleic acid synthesis and also function as regulatory molecules [32]. DNA methylation patterns which are largely established in-utero, induce stable changes in gene expression that may be sustained throughout the life span of an individual [33]. Associations of homocysteine with global methylation patterns are not well established. High concentrations of homocysteine have been reported to be associated with reduced DNA methylation potential by some [34], [35], while others have reported increased DNA methylation [36], [37]. On the other hand, recently Bromberg et al have reported no association between homocysteine and DNA methylation [38]. Our findings also suggest that homocysteine concentrations may not be the only determinant of global methylation levels in the placenta.

Low folic acid status is often associated with impaired DNA methylation [39], affecting gene expression in complex ways [40]–[43], however it is not known whether excess folic acid might have any adverse effects on these functions. In a recent study, Min et al.[44] have also shown that folic acid supplementation in vitamin B_12_ deficient rat did not alter hepatic SAM and SAH (S-adenosyl homocysteine) concentrations and DNA methylation. In our study, at normal folic acid there was no change in placental global DNA methylation levels. However, in contrast at excess folic acid levels in the absence of vitamin B_12_ it was lower as compared to control. It has been reported that effect of folate status on DNA methylation in animals and humans is tissue-, site-, and gene-specific [40], [43]. Our results suggest that it may be the ratio of folic acid and vitamin B_12_ that may play an important role in determining global DNA methylation.

Evidence from in vivo studies has not clearly established a link between vitamin B_12_ and DNA methylation. However it has been demonstrated in the animal model that a B_12_ deficient diet, disturbs normal homeostasis of one-carbon metabolism in the colonic mucosa and results in diminished genomic DNA methylation and increased uracil misincorporation in DNA [45]. Further, it has recently been shown that gene expression patterns change under B_12_ deficient conditions and are recovered by dietary methionine supplementation to B_12_ deficient rats [46].

For the first time this study has shown that supplementation with omega 3 fatty acids in excess folic acid and vitamin B_12_ deficient group increased placental global DNA methylation to control levels. During early development, there are two waves of demethylation which are followed by a gradual increase in de novo methylation in the embryonic and extraembryonic (which includes the placenta) tissues [47]. Deficiencies of vitamin B_12_ or other abnormalities within the one carbon cycle have been implicated in the development of such placental diseases [48]. Our findings suggest that placental maturation and development may be hampered due to vitamin B_12_ deficiency and absence of DHA leading to lower total global methylation (hypomethylation). DHA is reported to play an important role in development and maturation of vital tissues such as brain and placenta [49], [50]. Our findings indicate that DHA supplementation restores (increases) the global methylation levels to control levels suggesting that that omega 3 fatty acids especially DHA plays an important role in determining methylation levels in the placenta. Further, our results are in line with previous studies in the rat model which have demonstrated that supplementation with omega-3 fatty acids during pregnancy [51] or post natal life [52] could prevent or limit adverse outcomes of fetal programming. Further studies on gene-specific methylation involved in placental growth and pathology will throw light on the mechanisms to explain the current data.

The methodology for estimation of global methylation levels used in this study takes into account methylation of all CpG's irrespective of their position in the genome (promoter and non-promoter CpG). This is in concurrence with studies indicating that CpG methylation in intragenic and intergenic regions are also critical to gene expression [30]. Although this study has not examined methylation at gene specific level, our lab has initiated studies to examine the epigenetic changes occurring at genes associated with one carbon metabolism. Understanding these mechanisms may help in elucidating pathways associated with adverse pregnancy outcomes. In conclusion, changes in maternal micronutrients such as folate, vitamin B_12_ and omega-3 fatty acids could alter the availability of these key metabolites of one carbon cycle in the fetus, providing a direct link between maternal nutrition and placental gene methylation.
